# Overexpression of UHRF1 gene correlates with the major clinicopathological parameters in urinary bladder cancer

**DOI:** 10.1590/S1677-5538.IBJU.2016.0126

**Published:** 2017

**Authors:** Skender Saidi, Zivko Popov, Vesna Janevska, Sasho Panov

**Affiliations:** 1 University Clinic of Urology in Skopje, Republic of Macedonia;; 2 Institute of Pathology, Medical Faculty, Ss. Cyril and Methodius University, Republic of Macedonia;; 3 Molecular Biology and Genetics Department, Institute of Biology, Faculty of Natural Sciences and Mathematics, Ss. Cyril and Methodius University, Skopje, Republic of Macedonia

**Keywords:** Urinary Bladder Neoplasms, Carcinoma, Transitional Cell, RING Finger Domains

## Abstract

**Introduction:**

Recently, expression of the UHRF1 gene was found to be up-regulated in numerous neoplasms, including the urinary bladder transitional cell carcinoma (TCC).

**Objective:**

The aim of our study was to determine if the expression levels of UHRF1 gene correlates with the major pathological characteristics of the tumor and patients’ clinical outcome.

**Materials and Methods:**

In our study, we have analyzed the tissue samples derived from group of 70 patients with histologically confirmed TCC of the urinary bladder, while normal urinary bladder mucosa obtained from 40 patients with nonmalignant diseases was used as a negative control group. Expression of UHRF1 gene in each patient sample was determined using reverse transcriptase-polymerase chain reaction.

**Results:**

UHRF1 gene expression was found to be app. 2.5 times higher in samples from patients with TCC in comparison with normal epithelium derived from control group patients. Analysis show that gene expression correlates with the malignancy of the tumor. A highly significant differences were found between the expression values of samples from low and high grade TCC, as well as between the high grade and control group. UHRF1 expression was higher in patients with non-muscle invasive disease than in those with muscle invasive disease.

**Conclusions:**

The result of this study indicates that UHRF1 gene expression levels correlates with the major pathological characteristics of TCC samples and with the clinical outcome of those patients. Determination of UHRF1 gene expression could have a potential to be used as a sensitive molecular marker in patients with urinary bladder cancer.

## INTRODUCTION

Transitional cell carcinoma (TCC) of the urinary bladder cancer is a common malignancy in industrialized countries associated with high mortality thus having significant impact on public health (1-4). At the time of diagnosis, the majority of patients present with superficial neoplasm (restricted to the epithelium or the subepithelial connective tissue), while approximately 50% to 70% of patients develop disease recurrence with 10–40% of cases ultimately progressing to muscle invasive stage of advanced or metastatic disease over 5 years (5). Thus far, no validated and clinically useful molecular markers are established for urinary bladder TCC, as opposite to other urological cancers (6).

The Ubiquitin-like containing PHD Ring Finger 1 (UHRF1) protein, also called ICBP90, is a multidomain protein encoded by the UHRF1 gene that seems to have a complex transcription pattern. UHRF1 binds to specific DNA sequences, creates a complex with histone deacetylase 1 and DNA methyltransferase 1 and thus regulates gene expression. As a target of E2F transcription factor, UHRF1 facilitates the cell-cycle G1/S transition (7). In addition, UHRF1 downregulates the expression of several tumor suppressor genes including p16INK4A, hMLH1, BRCA1 and RB1 and functions in the p53-dependent DNA damage checkpoint during cell-cycle regulation. Some authors consider UHRF1 as a caretaker that has a critical role in the maintenance of the genome integrity (8).

Latter, it was described that UHRF1 gene expression is only detectable in proliferating cells, not in quiescent cells and that this gene is overexpressed in numerous malignant neoplasms including lung, breast, prostate, pancreatic and cervical cancer (9, 10). Some authors consider UHRF1 gene as an oncogene which overexpression leads to hypomethylation phenomenon in cancer genomes (11).

Recently, overexpression of UHRF1 gene was described in urinary bladder TCC, but conflicting results were obtained regarding the correlation and clinical significance of this molecular abnormality (12-14). Furthermore, it was demonstrated that the upregulated UHRF1 promotes bladder cancer cell invasion by epigenetic silencing of KiSS1 metastasis suppressor gene coding for kisspeptin (15).

The purpose of our study was to determine if the expression levels of UHRF1 gene in patients with TCC correlates with the major pathological characteristics of the tumor and patient’s clinical outcome.

## MATERIALS AND METHODS

### Patients and samples

‘In this study we analyzed tissue samples from 70 patients with histopathologically confirmed transitional cell carcinoma of the urinary bladder, collected by transurethral resection of bladder tumor at the University Clinic of Urology in Skopje between October 2009 and March 2011. Sixty-one male and 9 female patients with median age of 64.29±9.51 years (range 38-79) were included in the study. Clinicopathological parameters that were evaluated included: histopathological grade according to WHO 2004 classification system (LG and HG), pathological stage (non-muscle invasive: pTa, pTis or pT1, and muscle invasive: pT2 or higher), as well as clinical history (incidence of local recurrence, distant metastases and cancer-related death in the 2-year follow-up period).

Histological tumor grading and staging were considered at the time when the tissue sample was first obtained from the patient.

As a negative control patient group, tissue samples of histologically normal urinary bladder mucosa obtained by open retropubic prostatectomy for benign prostate hyperplasia (34 patients) or hysterectomy for nonmalignant purposes (6 patients) were used. The patients from the negative control group had age mean 64.24±12.58 years (range 34-81) and very similar gender distribution as those from the UBC group.

All patients signed written information consent and the study was approved by the Ethics Board of the Urology Clinic in Skopje (No 03-1165 from December 28, 2009).

### RNA isolation

Total cellular RNA was isolated from frozen tissue samples using TRI-reagent according to the manufacturer’s instructions. Residual DNA was digested by RNase-free DNase I at 37ºC for 30 min. After thermal inactivation of the enzyme at 95ºC for 5 min, the RNA was reprecipitated with isopropanol and rehydrated with RNase-free water containing 20 units of ribonuclease inhibitor. All above mentioned reagents were purchased from Sigma-Aldrich, UK.

### RT-PCR analysis

UHRF1 gene expression was measured by semiquantitative RT-PCR relative to housekeeping β-actin gene using reverse transcription system Enhanced Avian HS RT-PCR (Sigma-Aldrich, UK) following the manufacturer’s protocol. Oligo-dT primer was used for first-strand cDNA synthesis, while amplification of UHRF1 gene-specific mRNA transcripts was performed by primer pairs: forward, 5’-CCA GCA GAG CAG CCT CAT C-3’ and reverse, 5’-TCC TTG AGT GAC GCC AGG A-3’ with the following PCR program: initial denaturation at 94oC for 3 min, followed by 33 cycles of 94oC for 1 min, 60oC for 1 min and 68oC for 1 min, 45 secs, as described previously (16). Terminal extension at 68oC for 10 min was applied. Primer pair used for β-actin transcript was: 5’-GCT CGT CGT CGA CAA CGG CTC-3’ and 5’-CAA ACA TGA TCT GGG TCA TCT TCT C-3’. All oligonucleotide primers were ordered from Sigma-Genosys, UK). Each RT-PCR reaction was run in triplicates on GeneAmp System 2400 thermocycler (Perkin Elmer, USA) with control reaction with no RNA template included. Amplification products sizes were 74bp for UHRF1 and 311bp for β-actin gene transcript. After agarose gel electrophoresis and digital imaging, fluorescence intensity of the electrophoretic bands was quantified using the gel analysis function of ImageJ 1.48 software (Rasband, W.S., ImageJ, U. S. National Institutes of Health, Bethesda, Maryland, USA, http://imagej.nih.gov/ij/, 1997-2014). To quantify the relative levels expression of the target UHRF1, the value of the internal standard (β-actin) in each test tube was used as the background measurement (1.00) of gene expression in sample. The relative value of UHRF1 expression was calculated from ratio of the arbitrary units of the target gene to that of β-actin in the same PCR.

To determine the threshold value for overexpressed UHRF1 gene in our TCC patient’s group, the expression values form control mucosa samples were analyzed. As those values were consistently between 0.0803 and 1.3850 (mean 0.5826) ±standard deviation (1.866), values of 2.4484 (mean+10 standard deviations) or more were considered to show overexpression of the UHRF1 gene (17).

### Statistical analysis

Normal distribution of the gene expression data was evaluated by Shapiro-Wilk test. Correlations between the UHRF1 relative expression values from different patient’s groups were analyzed using non-parametric unpaired, two-tailed Mann-Whitney test comparing independent samples with unequal variance. P-values less than 0.05 and 0.01 were considered to be statistically significant and highly significant, respectively. Frequency of overexpressed UHRF1 gene was compared between patient’s subgroups by two-tailed Fisher exact test with calculations of Odds and Risk ratios at 95% confidence intervals. Statistical analyses were performed using Real Statistics Resource Pack software v. 4.3 on Microsoft Excel 2016.

## RESULTS

In this study, RT-PCR analysis revealed that the mean UHRF1 gene mRNA expression values in bladder cancer patients group were approximately 2.5 times higher than in the normal urothelium control group. Interestingly, the patients with solitary TCC has a significantly higher UHRF1 expression than the patients with multiple tumors (p<0.05).

**Figure 1:**
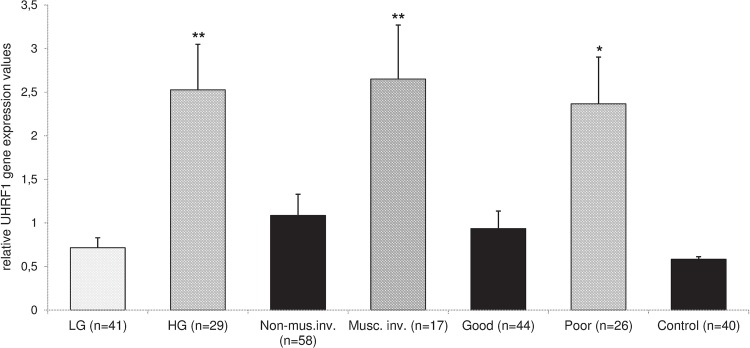
UHRF1 expression correlation with the T-classification grades, stages and the clinical outcome during 2-year follow-up of urinary bladder TCC patients.

We found that the obtained gene expression data correlates with the malignancy of the tumor (Figure-1). Namely, UHRF1 gene expression levels are lowest in control mucosa samples and in low grade TCC samples, but is significantly increased in the high grade samples (p<0.01, the exact p-values are given in Table-1). Statistical analysis revealed highly significant differences between the expression values from low and high grade samples, as well as between the high grade and the control group (p<0.01). On the opposite, the expression of UHRF1 gene was not significantly different between patients with TCC low grade and the control group with normal urinary bladder mucosa (p>0.05).

**LG and HG =** low and high grades, respectively; **Non-musc. inv. =** non-muscle invasive stage; **Musc. inv. =** muscle invasive (infiltrative) stage; **Good** outcome, no clinical signs of recurrence, metastasis or cancer-related death within the 2-year evaluation period; **Poor** clinical outcome indicates recurrence, metastasis or cancer-related death in the same period. **Columns**, means; bars, standard errors; *and**, p<0.05 and p<0.01, respectively, versus control.

**Table 1:** UHRF1 gene expression values regarding the demographic and selected clinicopathological parameters in urinary bladder TCC versus control patient’s samples and differences between the urinary bladder TCC subgroups.

Patients groups	Demographic and clinicopathological patient subgroups		Patients number n (%)	UHRF1 expression (mean±SE)	Difference vs. control group (p-value)	Difference between the two subgroups (p-value)
TCC	**Total**		**70 (100)**	**1.47±0.25**	**0.1826**	**/**

	Gender	Male	61 (87.14)	1.53±0.28	0.3090	0.4719
		Female	9 (12.86)	1.02±0.34	0.0814	
	Smoking history	No	23 (32.86)	1.56±0.48	0.2332	0.7546
		Yes	47 (67.14)	1.42±0.29	0.2664	
	Tumor number	Solitary	41 (58.57)	1.75±0.36	0.0348*	0.0927
		Multiple	29 (41.43)	1.06±0.30	0.9177	
	Tumor size (cm)	≤3	38 (54.29)	1.09±0.25	0.4268	0.4330
		>3	32 (45.71)	1.91±0.45	0.1275	
	Grades	Low	41 (58.57)	0.72±0.11	0.9060	0.0090**
		High	29 (41.43)	2.53±0.52	0.0057**	
	Stages	Non-muscle invasive	53 (75.71)	1.09±0.24	0.6222	0.0305*
		Muscle invasive	17 (24.29)	2.65±0.62	0.0087**	
	Outcome	Good	44 (62.86)	0.93±0.20	0.4654	0.1127
		Poor	26 (37.14)	2.37±0.54	0.0410*	

Control	**Total**		**40 (100)**	**0.58±0.03**	**/**	/

	Gender	Male	34 (85.00)	1.58±0.03	/	0.6359
		Female	6 (15.00)	1.60±0.05	/	

*and**, p<0.05 and p<0.01, respectively, versus control; **SE =** standard error.

Considering the histopathological staging categories, the expression value means were significantly lower in the non-muscle invasive than in the muscle invasive stage (p<0.05). There was a statistically highly significant difference between the muscle invasive stage TCC patient’s subgroup and control patients (p<0.01). However, the UHRF1 expression was not significantly higher in the non-muscle invasive stage than in the normal mucosa control samples (p>0.05).

Of the 70 analyzed patients, 26 had local tumor recurrences requiring multiple interventions, had a distant metastasis or died from this cancer within 2 years of obtaining the tissue sample. The mean expression values of UHRF1 in this poor outcome patient’s subgroup was significantly higher than in the control group (p<0.05). For the purpose of prognosis prediction, the frequency of patients with UHRF1 overexpressed above arbitrary estimated threshold value was analyzed instead of semiquantitative data. The UHRF1 gene was overexpressed in 10 patients (38.46%) out of the TCC subgroup with poor outcome during the 2-years follow-up in comparison with the 3 patients (6.82%) in the subgroup with good outcome (p=0.0016). The calculated Odds Ratio is 8.54 (at 95% confidence interval from 2.08 to 35.12) and Risk Ratio is 1.51 (at 95% confidence interval from 1.11 to 2.07).

No significant differences of UHRF1 expression values were found regarding the other demographic and clinicopathological parameters: gender, smoking history and tumor size.

## DISCUSSION

The overexpression of UHRF1 gene was recently described in many cancers, including urinary bladder TCC. However, although the expression levels were significantly higher in tumor samples than in the histologically normal urinary bladder, some authors did not identify any correlation between those values and the major pathological characteristics, the incidence of disease recurrence or patient’s survival (18). On the contrary, another study showed an association between UHRF1 gene expression and tumor recurrence in superficial bladder cancer of Chinese cases (13). Considering the relatively small number of studies published thus far, it is rather difficult to conclude the exact nature of those results discrepancies.

We examined the correlations between UHRF1 expression in urinary bladder TCC patients and selected demographic and clinicopathological parameters. The expression levels of UHRF1 correlated significantly with the WHO 2004 histological grading category HG, as well as with both stages (non-muscle invasive and muscle invasive), but no difference was found between low grade TCC and control samples, as well as between the non-muscle invasive stage and controls, suggesting that the UHRF1 gene expression correlated with the malignancy. Considering the mean expression levels were very similar in controls and in the low grade TCC samples, we hypothesized that UHRF1 overexpression initiates relatively late during the carcinogenesis and that this could be a consequence of a p53 dysfunction. Namely, some authors suggest that UHRF1 overexpression in cancer cells is partially due to p53 protein inactivation, since the last one is involved in UHRF1 gene regulation (13, 16). Previously, it was demonstrated that tumor-suppressor p53 protein indirectly downregulates UHRF1 expression by increasing the p21/WAF1 level and inactivation of E2F1 (19).

As experimentally induced UHRF1 gene knockdown in cancer cell lines leads to suppressed growth, the UHRF1 protein is essential for malignant cell’s proliferation and accordingly could be an attractive drug target (20). Furthermore, considering the estimated Risk Ratio (1.51), UHRF1 gene overexpression in TCC patients increases the risk of poor outcome for 51% in the 2-year evaluation period, compared to TCC subgroup with normal gene expression levels. Our results are in accordance with some of the previous studies about potential usefulness of UHRF1 overexpression as a prognostic prediction factor in urinary bladder cancer (12-14).

Moreover, our study confirms the importance of UHRF1 gene overexpression in urinary bladder epithelial carcinogenesis and further suggests that it could be used as a clinically useful molecular marker. Using of this molecular marker in combination with another one or additional parameters could potentially provide even greater sensitivity or patient’s outcome prediction.

The result of this study indicates that UHRF1 gene expression levels correlates with the high grade urinary bladder cancer, as well as with the muscle invasive stage. Further, results indicate that overexpression of this gene may predict the patient’s outcome in 2-years postoperative period. Determination of expression of UHRF1 gene could have a potential to be used as a very sensitive molecular marker with a prospective value in clinicopathological evaluation and prognosis of patients with urinary bladder cancer. More studies with larger patient’s groups and method validation are needed to confirm our results and establish stronger clinical usefulness of this molecular marker.
